# Anorexia Nervosa in U.S. Hispanic/Latino Populations: Sociocultural and Structural Influences on Recognition and Care

**DOI:** 10.3390/nu18213622

**Published:** 2026-10-31

**Authors:** Joanna Ortiz Martinez, Brianna Greene, Nhien Nguyen, Pei-An (Betty) Shih

**Affiliations:** Department of Psychiatry, University of California, San Diego, La Jolla, CA 92093, USA

**Keywords:** anorexia nervosa, Hispanic/Latino populations, prevalence, eating disorders, healthcare access, treatment utilization

## Abstract

**Background/Objectives**: Published U.S. studies have generally reported lower lifetime and 12-month prevalence of anorexia nervosa (AN) among Latino populations than among non-Hispanic White populations. Whether this disparity reflects epidemiologic differences or differences in detection and access to care remains unclear. We synthesized comparative prevalence estimates and examined factors that may influence their interpretation. **Methods**: We searched PubMed, Web of Science, and Google Scholar through August 2026 using PRISMA-guided study selection and conducted a random-effects meta-analysis of eight comparative studies. We also examined the findings in relation to diagnostic practices, sociocultural influences, and structural barriers to recognition and care. **Results**: Latino participants had lower odds of AN (pooled OR = 0.64, 95% CI = 0.49–0.82), with moderate between-study heterogeneity. Interpretation of this association is limited by differences in sampling and case ascertainment, unresolved heterogeneity across Latino subgroups, and limited data on Latino men. Additional considerations include diagnostic emphasis on low body weight, patterns of help-seeking, medical mistrust, stigma, acculturative stress, and structural barriers to care. Genetic studies of AN have drawn almost exclusively from European-ancestry cohorts, limiting their applicability to Latino populations. **Conclusions**: Lower reported AN prevalence among Latino populations may reflect a combination of epidemiologic differences and variation in sampling, symptom recognition, diagnosis, and access to treatment. Current biological evidence does not establish that AN is intrinsically less common among Latino populations. Future epidemiologic and genomic studies should recruit adequately powered, heterogeneous Latino samples. Clinical assessment should incorporate weight history and indicators of medical instability in addition to current body weight, alongside expanded availability of Spanish-language screening tools and bilingual, bicultural providers. Clinicians should remain alert to restrictive eating disorders across body sizes and cultural backgrounds.

## 1. Introduction

Eating disorders are serious psychiatric illnesses characterized by persistent disturbances in eating behavior and related thoughts and emotions. Anorexia nervosa (AN) involves restriction of energy intake, intense fear of weight gain or persistent behavior that interferes with weight gain, and disturbance in the experience or recognition of body weight or shape [1]. Pathological food restriction, compensatory behaviors, and severe psychopathology can lead to serious psychological and medical consequences and death. Reported lifetime prevalence estimates of AN range from 0.1% to 3.6% in women and from 0.1% to 0.3% in men [2,3,4,5]. AN commonly begins during adolescence [6] and disproportionately affects women, with a reported female-to-male ratio of 13:1 [5]. Although less prevalent than many other psychiatric disorders, AN is associated with substantial risk of relapse and elevated mortality, with reported standardized mortality ratios ranging from 1.2 to 15.9 [7,8].

Most AN research has disproportionately included White participants [9]. Consequently, the epidemiology, clinical presentation, illness course, and treatment outcomes of AN in other racial and ethnic populations remain incompletely characterized. Broader psychiatric and biomedical research has also documented racial and ethnic differences in disease prevalence, diagnosis, and treatment [10,11], underscoring the need to critically examine whether reported differences in AN prevalence reflect underlying differences in disorder occurrence, differential recognition and ascertainment, or both.

## 2. Methods

Study protocol overview: Because relatively few studies have examined AN specifically in Latino populations, we combined a structured review of prevalence studies, a quantitative meta-analysis, and a targeted narrative review to better understand reported differences in AN prevalence between Latino and non-Hispanic White populations. The structured literature search identified studies reporting AN prevalence or related case estimates among Hispanic/Latino populations, including comparisons across racial or ethnic groups. We followed PRISMA guidelines for study identification and selection. Eligible comparative studies were included in a random-effects meta-analysis to estimate the pooled odds ratio (OR) for AN in Hispanic/Latino versus White participants.

To address areas not adequately captured by prevalence studies, we also conducted a targeted narrative review of factors that may contribute to observed differences in AN prevalence, recognition, and diagnosis. Topics included the roles of BMI and weight history in AN diagnosis, help-seeking patterns, medical mistrust, stigma, cultural influences on weight- and eating-related factors, acculturative stress, and financial and structural barriers to care.

Because much of the available literature concerns women, the narrative discussion focuses primarily on Latina women. We use Hispanic/Latino to refer broadly to people of Latin American origin or Spanish-speaking cultural origin or heritage, consistent with classifications in the source studies. Hispanic/Latino ethnicity may coexist with any racial identity and is not synonymous with non-White race [12]. White comparison groups were classified as non-Hispanic White only when explicitly defined that way in the source study. We retained each study’s original classifications and did not create a separate “non-White Latino” category.

Literature search strategy and eligibility: We searched PubMed, Web of Science, and Google Scholar for peer-reviewed English- or Spanish-language studies available through August 2026. Full database-specific search strategies, final search dates, and filters or limits are provided in Table S3. Search terms included “anorexia nervosa,” “Hispanic,” “Latino,” “Latina,” “Latinx,” “prevalence,” “incidence,” “epidemiology,” “eating disorders,” and “racial/ethnic differences.” For Google Scholar, we screened the first 110 results ranked by relevance. Studies were eligible if they reported original data on AN prevalence or related case estimates among Hispanic/Latino participants. Studies that additionally reported AN prevalence in a White comparison group were eligible for inclusion in the meta-analysis.

**Figure 1:**
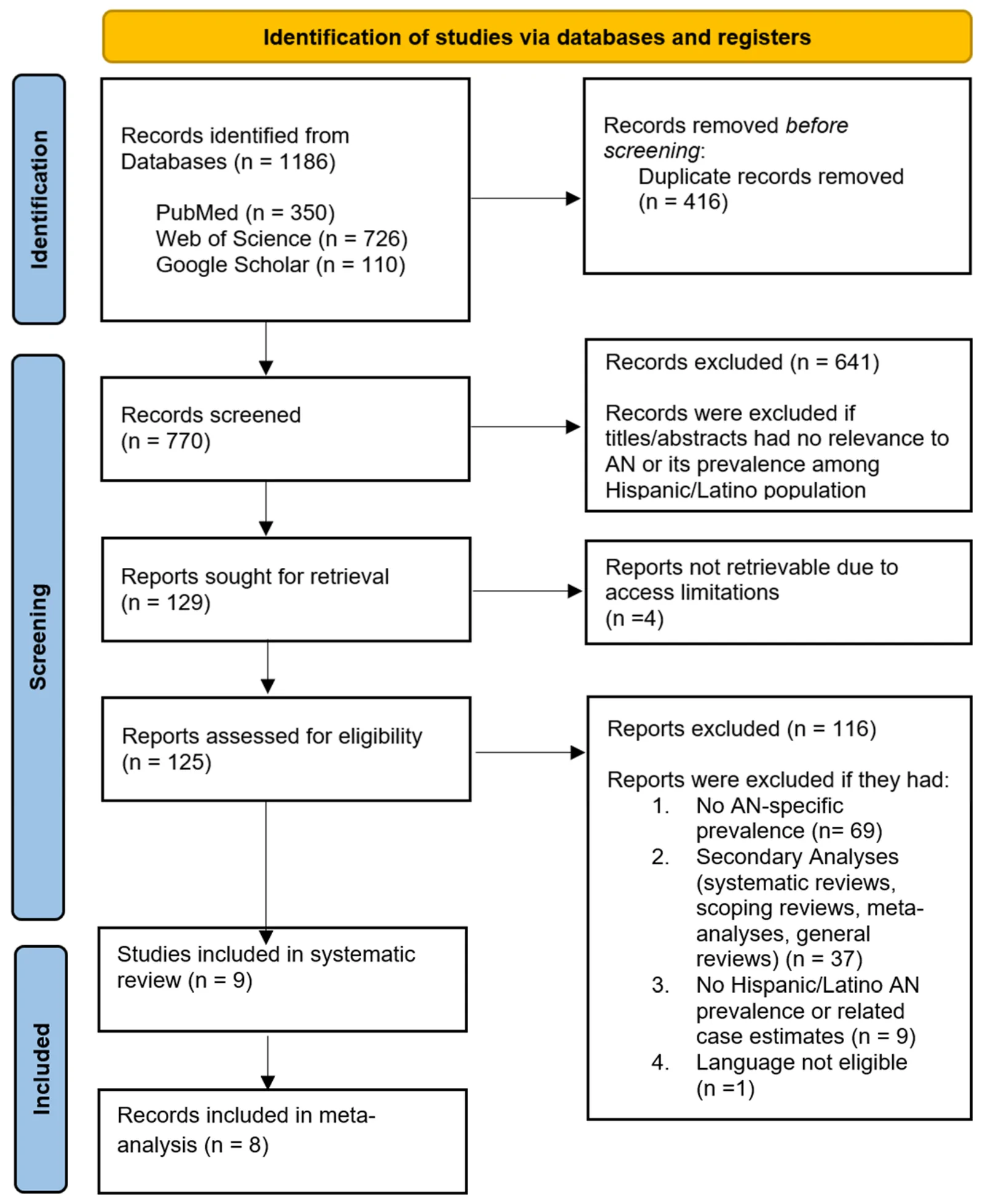
PRISMA 2020 flow diagram of the study selection process for studies reporting anorexia nervosa prevalence or related case estimates among Hispanic/Latino populations.

Selection process: After removal of duplicates, case reports, reviews, editorials, and non-peer-reviewed records, one author (J.O.) screened titles and abstracts and subsequently reviewed the full text of potentially eligible studies. B.G. served as a backup reviewer, independently verified the included studies and extracted information, and repeated the literature search using the same predefined keywords; no additional eligible studies were identified. J.O. extracted study characteristics and prevalence data, and N.N. independently verified the data used in the meta-analysis; discrepancies were resolved by consensus with P.B.S. Nine U.S. reports were included in the prevalence review, of which eight provided Hispanic/Latino–White comparisons and contributed to the meta-analysis. Alegría et al. reported Latino-only NLAAS findings that overlapped with the CPES sample analyzed by Marques et al. and did not contribute a separate effect estimate. The Mexico-based study by Benjet et al. is discussed for context and was not counted among the nine U.S. reports (Figure 1). Detailed sampling, diagnostic, and data-extraction characteristics are provided in Table S1.

Meta-analysis methods: We conducted a meta-analysis of eight studies using the metafor package (version 4.8-0) in R (version 4.5.1; R Foundation for Statistical Computing, Vienna, Austria) to compare the odds of AN among Hispanic/Latino participants with those among White participants. For studies reporting AN prevalence for multiple timeframes, lifetime prevalence was used when available to comprehensively capture AN occurrence. Outcome definitions were verified from the original publications, and estimates were extracted according to the terminology used by each study. For example, Cheng et al. (2019) [13] reported study prevalence, defined as participants meeting AN criteria at baseline or during follow-up, which was retained as the available prevalence estimate from that study. Howe et al. (2025) [14] assessed screening prevalence of probable AN using self-reported BMI and weight/shape concerns rather than lifetime diagnostic history. Study-specific odds ratios were calculated from case counts and corresponding sample sizes reported in the original publications or taken directly from published odds ratios. Confidence intervals displayed in the forest plot were computed during the meta-analysis.

Van Dyne et al. (2023) [15] reported only a 12-month AN estimate. Given the limited number of eligible comparative studies, we included this estimate to retain the available Hispanic/Latino–White comparative evidence, while recognizing that 12-month and lifetime prevalence represent distinct estimates. The potential influence of this study was evaluated in the leave-one-out sensitivity analysis.

Because the included studies differed in participant characteristics, study design, and sampling methods, a random-effects meta-analysis using restricted maximum likelihood (REML) was conducted on log odds ratios (ORs), with 95% CIs and a 95% prediction interval. Between-study heterogeneity was assessed using Cochran’s Q statistic, Higgins’ I^2^, and the estimated between-study variance (τ^2^). No zero cells occurred in the data used to calculate study-specific odds ratios; therefore, no continuity correction was required.

Sensitivity analyses included leave-one-out analyses, influence diagnostics, and the Knapp–Hartung adjustment to evaluate the robustness of the pooled estimate. Because only a small number of studies were included, Egger’s regression test was treated as exploratory.

This review/meta-analysis was not prospectively registered, and no formal protocol was developed prior to the study.

Given the small and heterogeneous evidence base, we did not assign a formal risk-of-bias score. Instead, we considered differences in sampling frame, representativeness, diagnostic ascertainment, and prevalence period when interpreting the pooled findings, and evaluated statistical robustness using leave-one-out analyses, influence diagnostics, and the Knapp–Hartung adjustment.

## 3. Results

### 3.1. Reported Prevalence of Anorexia Nervosa in Hispanic and Latino Populations

Across the included studies, reported AN prevalence was generally lower among Hispanic/Latino participants than among non-Hispanic White participants, although estimates varied across samples and assessment methods (Table 1). Additional AN estimates for other racial/ethnic groups reported in the included studies are presented in Table S2. Among the U.S.-based studies reporting overall lifetime prevalence, estimates ranged from 0.08% to 0.46% among Hispanic/Latino participants and from 0.39% to 0.96% among non-Hispanic White participants. Where sex-stratified estimates were reported, AN prevalence was generally higher among women than men. Estimates differed across studies in sampling, diagnostic criteria, ascertainment methods, and prevalence period (Table 1). The included studies were predominantly conducted in the United States and differed in participant age ranges, sampling strategies, diagnostic criteria, and assessment methods.

One Mexico-based study of adolescents aged 12–17 years reported a lifetime AN prevalence of 0.5% and a 12-month prevalence of 0.1% using DSM-IV criteria [21]. The authors noted several methodological limitations, including the use of trained lay interviewers, limited prior eating-disorder research for comparison, and reliance on self-reported height and weight. Although these estimates were of a similar order of magnitude to those reported in U.S.-based studies, such descriptive comparisons cannot establish whether AN prevalence differs between populations. We therefore conducted a meta-analysis to quantitatively compare the odds of AN among Hispanic/Latino and White participants across the eight studies that provided prevalence data for both groups.

### 3.2. Meta-Analysis Results

**Figure 2:**
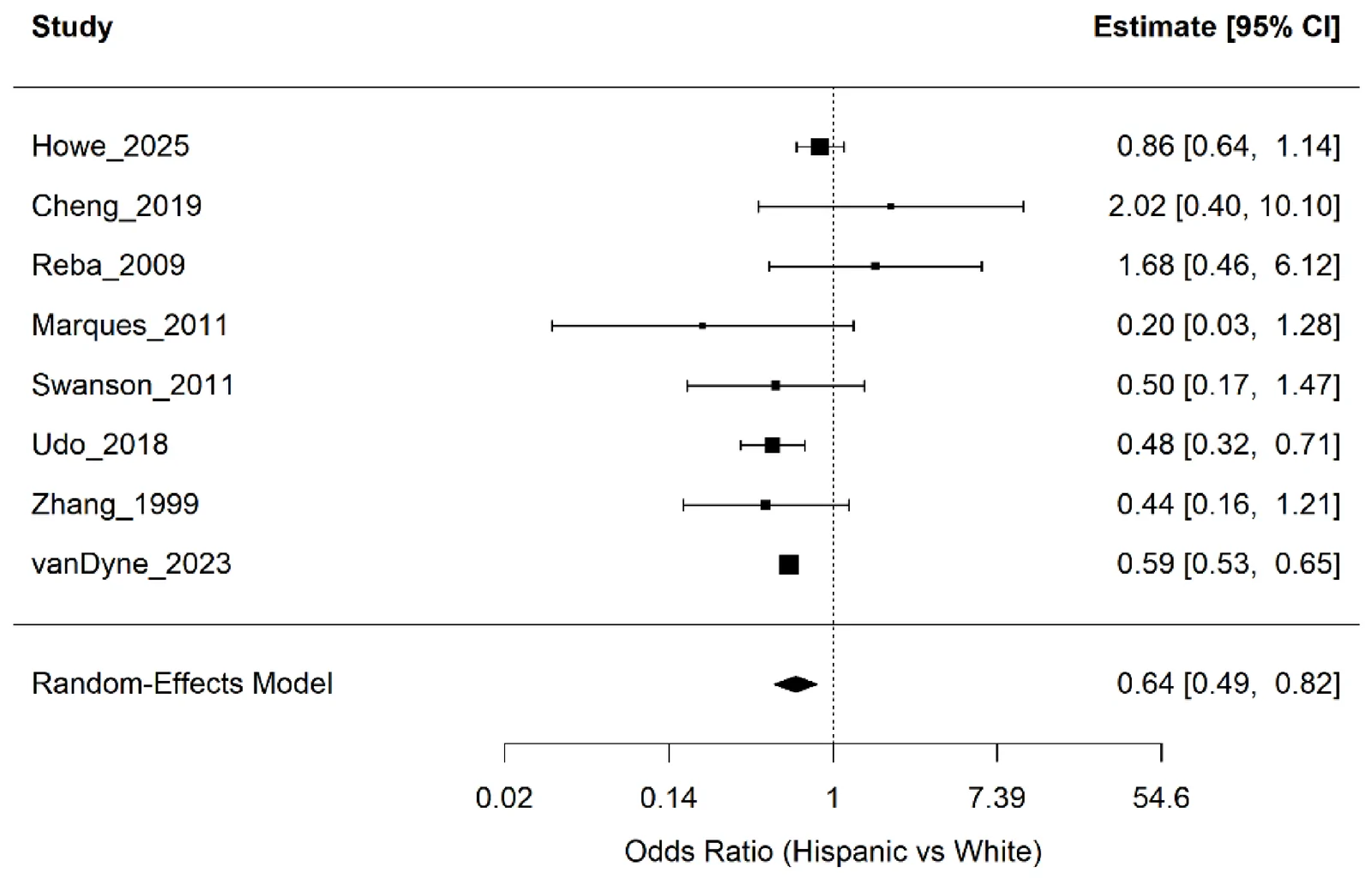
Forest plot showing odds ratios (OR) and 95% confidence intervals (CIs) for anorexia nervosa (AN) prevalence in Hispanic versus White populations. Each square represents the study-specific OR, and horizontal lines indicate the corresponding 95% CI. The diamond represents the pooled OR from the random-effects meta-analysis model. Included studies: Marques et al. [3], Cheng et al. [13], Howe et al. [14], Van Dyne et al. [15], Reba-Harrelson et al. [16], Swanson et al. [17], Udo and Grilo [18], and Zhang and Snowden [19]. Cheng et al. (2019) [13] contributed study prevalence across baseline and three-year follow-up; Howe et al. (2025) [14] contributed screening prevalence of probable AN; Reba-Harrelson et al. (2009) [16] contributed weighted prevalence of broadly defined AN with an unspecified recall period; and Van Dyne et al. (2023) [15] contributed past-year self-reported professional diagnosis or treatment. Lifetime prevalence estimates were used when available.

Across the eight studies [3,13,14,15,16,17,18,19], Hispanic/Latino participants had significantly lower pooled odds of AN than White participants (OR = 0.64, 95% CI = 0.49–0.82, *p* = 0.0005; Figure 2), corresponding to 36% lower odds. Between-study heterogeneity was moderate (Q = 13.60, df = 7, *p* = 0.059; I^2^ = 47.5%; τ^2^ = 0.044), indicating variation in the magnitude of the association across studies. The 95% prediction interval was 0.39–1.03, indicating that although the pooled OR is below the null, the true effect could plausibly be slightly above the null in a future setting.

Sensitivity analyses showed that the pooled association was generally stable. Leave-one-out estimates ranged from OR = 0.58 to 0.68 and remained statistically significant (*p* < 0.0001 to 0.03). Knapp–Hartung adjustment yielded directionally and statistically consistent results (OR = 0.64, 95% CI = 0.45–0.89, *p* = 0.014). Egger’s regression test was not significant (Z = 0.25, *p* = 0.80); however, given the small number of studies, this analysis had limited power to detect small-study effects.

## 4. Discussion

The meta-analysis showed lower reported odds of AN among Hispanic/Latino participants than among White participants. However, this finding does not establish whether the observed difference reflects variation in underlying disease occurrence, symptom presentation, case ascertainment, diagnostic recognition, or access to care. The heterogeneity of study types, reported prevalence, and outcome periods limits direct prevalence comparison across studies. Of the eight studies included in the meta-analysis, only Cheng et al. [13] and Reba-Harrelson et al. [16] yielded point estimates greater than 1 (Figure 2), indicating higher estimated odds of AN among Hispanic/Latino participants in those samples. In both studies, however, the confidence intervals included 1, and neither showed a statistically significant difference between groups. Cheng et al. analyzed participants enrolled in an eating-disorder prevention trial rather than a general-population prevalence sample and included substantially fewer Hispanic than White participants. Reba-Harrelson et al. [16] used a nonprobability national quota sample of U.S. women aged 25–45 years who voluntarily participated in an online survey. These design features limit the precision and generalizability of the resulting estimates.

Two large, frequently cited epidemiologic studies illustrate both the reported prevalence difference and important limitations of the available evidence. Analysis of National Latino and Asian American Study data identified only two women who met lifetime AN criteria among 2554 English- and Spanish-speaking Latino participants, corresponding to a lifetime prevalence estimate of 0.08% [20,22]. In NESARC-III, DSM-5 lifetime AN prevalence was 0.46% among Hispanic participants and 0.96% among non-Hispanic White participants; adjusted odds of AN were reported to be 52% lower among Hispanic participants [18]. However, only 36 Hispanic participants met criteria for AN, compared with 206 non-Hispanic White participants. Together, these findings are consistent with lower reported prevalence among Hispanic/Latino populations, while also illustrating that some estimates are based on relatively small numbers of Latino AN cases, which limits precision and the ability to characterize heterogeneity within this population.

A three-year prospective study of high-school- and college-aged women with body-image concerns found no statistically significant ethnic differences in the onset of threshold or subthreshold AN [13]. Because participants were selected on the basis of elevated eating-disorder risk rather than sampled from the general population, these findings cannot be interpreted as population-based estimates of AN incidence or prevalence. Nevertheless, the study demonstrates that threshold and subthreshold AN occur across racial and ethnic groups and reinforces the importance of applying appropriate screening and diagnostic assessment across diverse populations.

### 4.1. Interpreting Lower Reported Prevalence and Potential Under-Recognition

The available evidence supports a more cautious conclusion than either “AN is intrinsically uncommon among Latinos” or “the lower reported prevalence is entirely attributable to underdiagnosis.” The pooled analysis indicates lower reported odds of AN among Hispanic/Latino participants, but the limited number of studies, moderate between-study heterogeneity, relatively small numbers of Latino AN cases in several studies, and differences in sampling and assessment leave the explanation for this disparity unresolved. Lower observed prevalence could reflect differences in underlying disease occurrence, case identification, or both.

The following sections therefore examine factors that could contribute to differential recognition or ascertainment of AN among Latino populations. These include diagnostic emphasis on low current BMI, differences in help-seeking, medical mistrust, stigma, cultural influences on eating and weight-related attitudes, acculturative stress, and financial, linguistic, and structural barriers to care. These factors should be considered as potential explanations for the observed epidemiologic pattern rather than as established causes of the lower reported prevalence.

### 4.2. BMI, Weight History, and Potential Diagnostic Under-Recognition

Body mass index (BMI; kg/m^2^) informs the assessment of low weight and the DSM-5 severity specifier for AN, but it is not the sole diagnostic criterion. DSM-IV described body weight below 85% of that expected as an example of significantly low weight rather than an absolute diagnostic threshold, although this benchmark was frequently operationalized in research and clinical practice [23]. DSM-5 removed the amenorrhea criterion, revised the low-weight criterion, and introduced adult severity categories based on BMI: mild (BMI ≥ 17 kg/m^2^), moderate (16.00–16.99 kg/m^2^), severe (15.00–15.99 kg/m^2^), and extreme (<15 kg/m^2^) [24,25]. These categories characterize severity partly on the basis of current body weight but do not, by themselves, capture premorbid weight, magnitude or rate of weight loss, or physiologic instability.

Changes from DSM-IV to DSM-5 increased case identification in some populations. In a Finnish twin cohort, 55 women met lifetime DSM-IV criteria for AN, whereas an additional 37 cases were identified using DSM-5 criteria, increasing estimated lifetime prevalence from 2.2% to 3.6% [26]. This increase reflected multiple diagnostic changes, including removal of the amenorrhea criterion and modification of the low-weight criterion, and therefore cannot be attributed to BMI alone. More broadly, reliance on current body weight may fail to adequately characterize illness severity in individuals who experience substantial weight loss from a higher premorbid weight.

Evidence from other areas of medicine illustrates that the relationship between BMI and health risk is not necessarily uniform across populations. A WHO expert consultation found that some Asian populations have greater body fat and increased risk of type 2 diabetes and cardiovascular disease at lower BMI values than White or European populations of the same age and sex [27]. The consultation retained the existing international BMI classifications but identified lower BMI values as potential public-health action points. Subsequent work has similarly demonstrated population differences in the relationship between BMI, adiposity, and metabolic risk [28]. Importantly, these findings concern overweight, adiposity, and metabolic disease and do not establish that ethnicity-specific BMI thresholds should be used to diagnose AN. Rather, they illustrate a broader limitation of assuming that a single BMI value has identical clinical meaning across populations.

Higher average BMI and a higher prevalence of overweight or obesity have been reported in U.S. Hispanic/Latino populations compared with non-Hispanic White populations [29,30,31]. While these population-level observations should not be interpreted as inherent biological differences or applied to individual patients, their relevance to AN is more specific: an individual beginning at a higher premorbid weight can undergo substantial and rapid weight loss, develop malnutrition and medical instability, and still remain above a conventionally low BMI. Indeed, some studies have found that even loss of 5% of body weight can produce clinically significant physical and psychological symptoms in normal-weight individuals [32]. Assessment of restrictive eating disorders should therefore incorporate weight history, magnitude and rate of weight loss, restrictive behaviors, eating-disorder cognitions, and physiologic status rather than relying on current BMI alone.

The applicability of this evidence to Hispanic/Latino populations remains uncertain because racial and ethnic diversity has been poorly characterized in the atypical AN literature; in one systematic review, only 36% of studies reported race and 19% reported Hispanic ethnicity, with most samples predominantly White [33]. Under-recognition of atypical AN may further contribute to missed or delayed diagnosis, particularly when substantial weight loss occurs without a conventionally low BMI, as clinicians are less familiar with atypical AN and less likely to identify, counsel, or refer affected patients [34]. Together, limited representation in the literature, reliance on current BMI, and potential under-recognition of clinically significant weight loss may contribute to incomplete case ascertainment and, consequently, underestimation of AN prevalence in Hispanic/Latino populations.

Taken together, these patterns suggest a plausible, though currently untested, hypothesis: because standard AN diagnostic criteria are most likely to miss cases presenting at higher body weight, and because Hispanic/Latino populations in the United States have a higher average BMI than non-Hispanic White populations [29,30,31], atypical AN could be relatively more common, rather than less common, among Hispanic/Latino individuals than the standard-AN prevalence gap alone would suggest. No study to date has directly examined atypical AN prevalence by race or ethnicity with adequate power, so this remains speculative. Future research should investigate further the prevalence of atypical versus typical AN in ethnic minority groups to better ascertain whether the reported prevalence gap is reflective of a true absence of restrictive eating pathology in this population.

### 4.3. Help-Seeking Behavior Among Hispanic/Latino Individuals

Lower rates of help-seeking and treatment utilization may contribute to the comparatively low reported prevalence of AN among Hispanic/Latino populations by reducing opportunities for case identification. Using data from the 2012–2013 National Epidemiologic Survey on Alcohol and Related Conditions-III, one study examined help-seeking for eating disorders among U.S. adults assessed with the AUDADIS-5. For AN, respondents were asked whether they had ever sought help related to low weight through professional care, support groups, hospitalization, emergency department visits, prescribed medication, or 12-step programs. After adjustment for sociodemographic characteristics, Hispanic/Latino respondents with AN were significantly less likely than non-Hispanic White respondents to seek treatment (OR = 0.30, 95% CI [0.13–0.67], *p* < 0.01), including care from counselors or psychologists and other forms of mental-health treatment [35].

Similarly, a study of Mexican American (MA) and European American (EA) women found that although the two groups were equally likely to recognize a significant eating problem and to report wanting help, fewer MA women sought treatment (27.6% vs. 44.9%). EA women were also more likely to receive eating-disorder treatment (61.3% vs. 23.8%), to receive care from psychotherapists or psychiatrists, and to be prescribed psychotropic medication, whereas MA women were more likely to receive prescription diet pills. Notably, among all women who sought professional help, only 47% received an eating-disorder diagnosis or treatment, with missed opportunities for detection and treatment particularly evident among MA women [36].

Broader patterns of mental-health service utilization are consistent with these findings. Alegría et al. reported specialty mental-health service use among 5.9% of Latino participants compared with 11.8% of non-Latino White participants [37], and other studies have identified barriers to mental-health care among low-income Latina immigrants [38]. Although these studies do not directly quantify missed AN diagnoses, they support a plausible pathway through which Hispanic/Latino individuals with eating-disorder symptoms may be less likely to reach settings where AN is recognized and documented.

These barriers may reduce diagnoses documented in clinical settings or reported as prior professional diagnoses. Their relevance to community surveys using standardized diagnostic interviews is less direct, because those interviews can identify AN regardless of prior treatment. Differences in help-seeking therefore cannot be assumed to explain the pooled prevalence difference.

### 4.4. Medical Mistrust and Mental Health Stigma

Medical mistrust may further reduce engagement with mental-health and eating-disorder services among some Hispanic/Latino individuals. Studies of Latino adults have linked perceived discrimination, language barriers, and lack of insurance with greater mistrust and reduced access to care [39,40,41,42]. Evidence of implicit bias among health professionals also raises concerns about communication and diagnostic quality [41]. Although these studies are not specific to AN, they identify factors that could reduce opportunities for timely recognition and assessment of restrictive eating disorders like AN.

Immigration-related concerns may create additional barriers for some Latino patients, particularly those who are undocumented. In a qualitative study of undocumented Latino immigrants and service providers in the United States, participants described fear, policy-related stress, and avoidance of nonemergency healthcare [43]. These findings should not be generalized to all Hispanic/Latino populations, but they illustrate how structural barriers may further limit contact with healthcare settings in which eating disorders might otherwise be identified.

Mental-health and eating-disorder stigma may also discourage symptom disclosure and professional help-seeking. Qualitative accounts from Latina women have described limited family awareness of eating disorders, shame, and beliefs that AN does not occur in Latina communities [44]. Research across racial and ethnic groups also suggests that some individuals turn first to family members rather than professional services [45]. Family support can be beneficial, but limited knowledge of eating disorders may delay recognition and referral. More broadly, eating-disorder stigma has been associated with poorer psychological outcomes and less favorable attitudes toward help-seeking [46,47].

Religious and spiritual coping may provide important support for some Hispanic/Latino individuals and should not be viewed as inherently obstructive. However, when mental-health symptoms are interpreted solely through a religious framework or spiritual responses substitute for clinical assessment, help-seeking may be delayed [12]. Taken together, mistrust, structural barriers, stigma, and culturally shaped pathways to care may reduce opportunities for AN recognition and documentation, potentially contributing to incomplete case ascertainment in Hispanic/Latino populations.

### 4.5. Complimentary Weightism and Weight-Related Comments

Though criticism of appearance has been substantially linked to body image dissatisfaction, comments intended as compliments about weight or shape can likewise increase body surveillance, dissatisfaction, anxiety, or self-objectification. This phenomenon has been described as complimentary weightism. In one ethnically diverse sample, Latina women reported more negative responses to appearance-related comments than White women [48]. Because the evidence is limited and context-dependent, it does not establish a general ethnic vulnerability. Clinically, the relevant point is that praise for weight loss may reinforce restrictive behavior or delay recognition of illness in any patient.

Weight-related comments from family and significant others may also be relevant. Eisenberg et al. reported that 43.5% of Latina young adults, compared with 28.6% of European American women, had received negative weight comments from family members [49]. Such cross-sectional differences do not establish that comments cause later development of AN, but they support asking patients about the social context of weight and shape concerns rather than assuming that thinness-related pressures operate uniformly across racial groups [50].

### 4.6. Acculturative Stress and Thin-Ideal Internalization

Eating-disorder symptoms and the social meanings attached to weight and shape may vary within and across cultural groups. Acculturative stress refers to psychological and social strain associated with adapting to a different cultural environment, including language barriers, discrimination, and conflict between cultural expectations [51]. In Latino college samples, greater acculturative stress has been associated with body-image disturbance and eating-disorder symptoms [51,52]. These findings are largely cross-sectional and therefore do not establish that acculturative stress causes AN or explains ethnic differences in AN prevalence.

Thin-ideal internalization refers to the extent to which an individual adopts socially valued thinness as a personal standard and is an established risk factor for eating-disorder symptoms and pathology [53]. Cultural adaptation may alter exposure to, or endorsement of, prevailing body ideals, but acculturation should not be conceptualized as a simple linear process or as uniformly harmful. Future longitudinal studies using culturally validated measures should examine whether acculturative stress and thin-ideal internalization, independently or interactively, influence AN risk, symptom presentation and reporting, or help-seeking among Hispanic/Latino populations.

### 4.7. Financial and Social Barriers

Financial and structural barriers may further limit access to eating disorder assessment and treatment. In the United States, specialized eating-disorder care can require substantial out-of-pocket expenditure, particularly when higher levels of care are needed. Project HEAL reports approximate costs of $150 per outpatient session, $1500 per week for intensive outpatient treatment, and up to $2000 per day for residential or inpatient treatment; the organization also cites a UC San Diego estimate of approximately $80,000 for an average eating-disorder treatment episode [54]. These costs are substantial relative to a U.S. median household income of approximately $80,610 in 2023 [55,56].

Financial burdens associated with treatment may not be distributed equally across populations. In 2023, median household income was approximately $84,630 among White households versus $65,540 among Hispanic households [55,56]. These aggregate differences cannot determine an individual family’s ability to pay for care, but they suggest that high out-of-pocket costs and limitations in insurance coverage may impose a greater relative financial burden on some Hispanic/Latino families. Material hardship may compound these barriers: using data from the 2021 Survey of Income and Program Participation, Scherer and colleagues reported that 34.8% of Hispanic individuals experienced food, housing, or bill-paying hardship in 2020, compared with 24.3% of non-Hispanic White individuals [57]. Insurance coverage also varies across racial and ethnic groups [58]. Together, these circumstances may reduce the financial, transportation, and scheduling flexibility required to obtain specialized assessment and treatment.

Additionally, even insurance coverage does not ensure access to eating-disorder care. Authorization for treatment may be denied or discontinued when patients do not meet an insurer’s medical-necessity criteria, including criteria that incorporate weight or other indicators of illness severity [59,60,61]. Patients who have experienced substantial weight loss or medical instability but remain at a higher BMI may therefore encounter barriers to authorization for higher levels of care. Whether the frequency or consequences of such denials differ systematically by race or ethnicity remains insufficiently studied and warrants further investigation.

Public insurance may also provide limited practical access when few specialized eating-disorder providers participate in Medicaid. In one 2022 assessment, only 3 of 64 clinicians certified in Family-Based Treatment reportedly accepted Medicaid [62]. Although this finding cannot be generalized to all eating-disorder providers, it illustrates a potential mismatch between insurance coverage and availability of specialized care. Geographic scarcity and transportation requirements may create additional barriers, particularly for intensive outpatient and partial-hospitalization programs that require frequent attendance. Telehealth can expand access to some forms of outpatient care but cannot fully replace in-person medical monitoring or higher levels of treatment. Collectively, these financial, insurance, and geographic constraints may reduce opportunities for timely diagnosis and treatment, particularly in underserved communities. Additionally, as many eating disorder studies are conducted out of these treatment facilities, these disparities in access to treatment may represent another factor impeding representation of Hispanic/Latino populations in research.

## 5. Limitations and Future Directions

The review process also had limitations. Initial title/abstract and full-text screening was conducted by one reviewer. Although a second reviewer independently verified included studies and repeated the search, this did not constitute independent duplicate screening of all records. Restriction to English- and Spanish-language publications and screening only the first 110 Google Scholar results ranked by relevance may have resulted in missed eligible studies. The search was limited to PubMed, Web of Science, and Google Scholar and did not include PsycINFO, Embase, Scopus, CINAHL, LILACS, or SciELO. The English- and Spanish-language restriction also excluded Portuguese-language publications. These restrictions may have resulted in missed relevant studies, including research involving Brazilian-origin populations, and limit the completeness of the review.

The pooled analysis combined studies differing in sampling frame, diagnostic criteria, age and sex composition, and outcome ascertainment, including diagnostic interviews, screening, and self-reported professional diagnosis or treatment. The pooled OR therefore summarizes associations across heterogeneous study settings rather than a uniform diagnostic outcome or a population-representative prevalence difference. Random-effects modeling and leave-one-out analyses do not eliminate these differences.

Several limitations in the existing literature constrain the specificity of the conclusions that can currently be drawn, and each identifies a concrete direction for future research. First, the studies available for meta-analysis did not consistently report prevalence by country of origin, so our pooled estimate necessarily treats Hispanic/Latino participants as a single group despite well documented heterogeneity in ancestry, migration history, and cultural context across Mexican, Caribbean, Central American, and South American populations [12]. This is a limitation of the underlying evidence base rather than of the present synthesis, and it underscores the need for future prevalence and treatment studies adequately powered to report country of origin specific estimates rather than a single aggregated Hispanic/Latino category. In addition, although lifetime prevalence estimates were preferentially selected for meta-analysis, prevalence definitions may differ across studies (e.g., study prevalence incorporating baseline cases and cases identified during follow-up). The included studies also varied in diagnostic criteria (DSM-III, DSM-IV, and DSM-5) and case ascertainment methods (diagnostic interviews, validated screening instruments, and self-reported diagnoses). Future epidemiologic studies using standardized diagnostic definitions and ascertainment methods would improve the comparability and precision of prevalence estimates across racial and ethnic groups.

Second, although several included studies reported male-specific prevalence estimates, evidence on AN among Latino men remains limited. Small numbers of identified cases and inconsistent sex-specific reporting constrained our ability to evaluate whether the Hispanic/Latino–White association differs by sex. Future studies should recruit sufficient numbers of Latino men to characterize prevalence, clinical presentation, and access to care.

Third, large scale genetic studies of AN, including Psychiatric Genomics Consortium eating disorder GWAS efforts, have drawn almost exclusively from cohorts of European ancestry across 17 countries [63]. Consequently, the genetic architecture and heritable liability of AN in Latino populations remain largely uncharacterized rather than demonstrated to be lower. Any biological interpretation of the observed prevalence disparity is therefore constrained by an absence of ancestry-diverse genomic data, and inclusion of Latino participants in future GWAS consortia is needed before genetic contributions to this disparity can be meaningfully evaluated.

Finally, these evidentiary gaps have direct clinical implications. Given documented barriers related to language and culturally specific presentation, expanding the availability and validation of Spanish language screening instruments and diagnostic interviews, along with increasing access to bilingual and bicultural mental health providers, may meaningfully improve case identification and engagement among Latino patients in the United States and should be prioritized in future clinical and health services research.

## 6. Conclusions and Perspectives

Taken together, the available evidence indicates that the lower reported prevalence of anorexia nervosa among Hispanic/Latino populations should be interpreted cautiously rather than assumed to reflect a genuinely lower underlying burden of disease. Although our meta-analysis found 36% lower pooled odds of AN among Hispanic/Latino compared with White participants (OR = 0.64, 95% CI 0.49–0.82), the available studies were heterogeneous and often included relatively small numbers of Hispanic/Latino participants with AN. Consequently, it remains unclear to what extent this disparity reflects true differences in disease occurrence versus differences in symptom presentation, case ascertainment, diagnostic recognition, and access to care.

Across the literature reviewed here, the factors described in the preceding sections could plausibly reduce opportunities for AN detection in Hispanic/Latino populations. None of these factors have been demonstrated to account for the observed prevalence difference on its own, and Hispanic/Latino populations should not be treated as culturally or clinically homogeneous; rather, these findings identify points along the pathway from symptom development to diagnosis at which cases may be overlooked or care delayed. These observations support greater race- and ethnicity-informed vigilance across the continuum of care.

Greater representation of Hispanic/Latino individuals in epidemiologic, clinical, and treatment research—together with the methodological priorities outlined above—will be essential to distinguish true epidemiologic variation from differences in symptom recognition, referral, diagnosis, and treatment access. Future studies should also examine where disparities emerge along the clinical pathway and whether culturally adapted approaches improve engagement and outcomes. Ultimately, improving case detection and ensuring equitable, culturally responsive assessment and treatment may both clarify the epidemiology of AN in Hispanic/Latino populations and, more importantly, reduce preventable delays in diagnosis, facilitate earlier intervention, and improve opportunities for sustained recovery.

## Figures and Tables

**Table 1:** Study Characteristics and AN Prevalence in Hispanic/Latino and White Populations.

Study	Population and Sampling Design	Age and Sex/Gender	Case Ascertainment and Diagnostic Criteria	Outcome Type and Prevalence Period	Hispanic/Latino Estimate	White Estimate	Between-Group Comparisons (Effect Estimate, 95% CI, *p* Value)
Cheng et al., 2019 [13]	Combined data from 3 U.S. ED prevention trials; female high school and college participants	Mean age 18.6 years (SD 4.3); women only	Eating Disorder Diagnostic Interview (EDDI); DSM-5; semi-structured diagnostic interview	Study prevalence across baseline and 3-year follow-up	**2/141 (1.4%)**	**6/848 (0.7%)**	3-year AN onset, Hispanic American vs. White (ref.): HR = 0.57 (95% CI 0.19–1.77); *p* = 0.332.
Howe et al., 2025 [14] ^#^	Population-based online mental health screening of students from 26 U.S. colleges	Mean age 20.2 years (SD 4.0); cisgender women, cisgender men, and non-cisgender participants	Stanford-Washington Eating Disorder Screen (SWED); probable DSM-5 AN (BMI ≤ 18.45 and WCS ≥ 59); self-reported screening	Screening prevalence of probable AN	Cisgender Women: 48/2585 (1.86%); Cisgender Men: 5/1065 (0.47%); Non-cisgender: 3/125 (2.40%); Total: **56/3775 (1.48%)**	Cisgender Women: 243/10,072 (2.41%); Cisgender Men: 21/5133 (0.41%); Non-cisgender: 9/590 (1.53%); Total: **273/15,795 (1.73%)**	No statistical detail reported for Latino–White comparison.
Marques et al., 2011 [3]	Nationally representative U.S. adults (CPES: NLAAS, NSAL, NCS-R)	Adults ≥ 18 years; men and women	World Mental Health Composite International Diagnostic Interview (WMH-CIDI); DSM-IV; structured interview	Lifetime weighted prevalence	**All 0.08%**; female: 0.12%; male: 0.03%	**All 0.39%;** female: 0.64%; male: 0.14%	(Latino vs. White) All: *p* = 0.0363; female: *p* = 0.0518; male: *p* = 0.6977
				12-month weighted prevalence ^†^	All: 0.03%; female: 0.02%; male: 0.03%	All: 0.03%; female: 0.02%; male: 0.04%	(Latino vs. White) All: *p* = 0.9985; female: *p* = 0.9937; male: *p* = 0.9955
Reba-Harrelson et al., 2009 [16]	U.S. women ages 25–45 recruited through a national quota-sampling procedure; non-probability online sample (N = 4023)	Mean age 35.2 years (SD 5.9); women only; ages 25–45	Online diagnostic questions modeled on the Structured Clinical Interview for DSM (SCID)/DSM-IV; broad AN defined as BMI ≤ 18.5 without requiring amenorrhea; self-report survey	Weighted prevalence of broadly defined AN; recall period not explicitly specified	**Weighted prevalence: 0.6%**	**Weighted prevalence: 0.3%**	No statistical detail reported for Latino–White comparison.
Swanson et al., 2011 [17]	Combined household and school-based adolescent samples from the National Comorbidity Survey–Adolescent Supplement (NCS-A; N = 10,123)	Adolescents ages 13–18 years; boys and girls	Modified World Health Organization Composite International Diagnostic Interview (CIDI); DSM-IV; face-to-face interviews administered by lay interviewers	Lifetime prevalence	**0.2%**	**0.4%**	No statistical detail reported for Latino–White comparison.
Udo & Grilo, 2018 [18]	Nationally representative U.S. adult sample from the National Epidemiologic Survey on Alcohol and Related Conditions-III (NESARC-III; N = 36,306)	Adults ≥ 18 years; men and women	Alcohol Use Disorder and Associated Disabilities Interview Schedule-5 (AUDADIS-5); DSM-5; computer-assisted face-to-face structured diagnostic interview; lowest self-reported BMI < 18.5	Lifetime weighted prevalence	36 cases (**0.46%)**	206 cases **(0.96%)**	Hispanic vs. non-Hispanic White (ref.) AOR = 0.48 (95% CI 0.33–0.72); *p* < 0.01
				12-month weighted prevalence ^†^	1 case (0.01%)	11 cases (0.07%)	Hispanic vs. non-Hispanic White (ref.) AOR = 0.11 (95% CI 0.01–1.00); *p* < 0.05
Zhang & Snowden, 1999 [19]	Community residents from 5 U.S. Epidemiologic Catchment Area (ECA) sites; Los Angeles site analyzed separately	Adults ≥ 18 years; men and women	Diagnostic Interview Schedule (DIS); DSM-III; highly structured interview administered by nonprofessional interviewers	Lifetime weighted prevalence	Full sample: **0.4%**	Full sample: **0.9%**	Hispanic vs. White (ref.) AOR = 0.385 (95% CI 0.140–1.062); *p* value not reported
Alegria et al., 2007 [20] *	Nationally representative U.S. Latino adult household sample from the National Latino and Asian American Study (NLAAS; N = 2554)	Adults ≥ 18 years; men (n = 1127) and women (n = 1427); English- and Spanish-speaking participants	World Mental Health Composite International Diagnostic Interview (WMH-CIDI); DSM-IV; fully structured diagnostic interview conducted in English or Spanish	Lifetime Bayesian prevalence	Overall: 0.08%; women: 0.12%; men: 0.03%	Not reported	Not Applicable: No comparison group
				12-month Bayesian prevalence	Overall: 0.03%; women: 0.02%; men: 0.03%	Not reported	Not applicable: No comparison group
Van Dyne et al., 2023 [15]	U.S. college students who voluntarily completed the American College Health Association–National College Health Assessment II-C (ACHA-NCHA II-C) survey	Mean age 22.5 years (SD 6.091); 68.5% female	Survey item asking whether a professional diagnosed or treated the student for anorexia within the past 12 months; no DSM diagnostic interview reported	Past-year self-reported professional diagnosis/treatment	Odds ratio only	Odds ratio only	Hispanic/Latino vs. White (ref.) **OR = 0.586** (95% CI 0.527–0.651); *p* < 0.001

Note. Race/ethnicity and sex/gender classifications were retained as defined in the original source studies. Categories may differ across studies in their definitions. Sex and gender classifications were retained as reported in the original studies and should not be interpreted as directly equivalent. Outcome periods and case ascertainment methods varied across studies. Abbreviations: AOR: adjusted odds ratio; HR = hazard ratio; CI = confidence interval; ref. = reference group; ACHA-NCHA II-C = American College Health Association–National College Health Assessment II-C; AUDADIS-5 = Alcohol Use Disorder and Associated Disabilities Interview Schedule–5; CIDI = Composite International Diagnostic Interview; CPES = Collaborative Psychiatric Epidemiology Surveys; DSM-III = Diagnostic and Statistical Manual of Mental Disorders, Third Edition; DSM-IV = Diagnostic and Statistical Manual of Mental Disorders, Fourth Edition; DSM-5 = Diagnostic and Statistical Manual of Mental Disorders, Fifth Edition; ECA = Epidemiologic Catchment Area Study; EDDI = Eating Disorder Diagnostic Interview; NCS-A = National Comorbidity Survey Replication–Adolescent Supplement; NCS-R = National Comorbidity Survey Replication; NESARC-III = National Epidemiologic Survey on Alcohol and Related Conditions III; NLAAS = National Latino and Asian American Study; NSAL = National Survey of American Life; SWED = Stanford-Washington University Eating Disorders Screen; WMH-CIDI = World Mental Health Composite International Diagnostic Interview. * Not included in the meta-analysis because a White comparison group was unavailable. ^†^ Twelve-month estimate not used when a lifetime estimate from the same study was available. **^#^** For Howe et al. (2025) [14], Table 1 includes only participants with reported gender; Table S1 lists the full racial/ethnic samples. **Bold** indicates estimates/data used in the meta-analysis.

## Data Availability

The original contributions presented in this study are included in the article/Supplementary Material. Further inquiries can be directed to the corresponding authors.

## Supplementary Materials

The following supporting information can be downloaded at: https://www.mdpi.com/article/10.3390/nu18213622/s1, Table S1: Detailed sampling, diagnostic, and data-extraction characteristics of included studies; Table S2: Additional racial/ethnic anorexia nervosa estimates reported in included studies; Table S3: Database-specific search strategies, final search dates, and filters/limits.

## References

[1] Mayo Clinic Staff Anorexia Nervosa. https://www.mayoclinic.org/diseases-conditions/anorexia-nervosa/symptoms-causes/syc-20353591.

[2] Hudson J.I., Hiripi E., Pope H.G., Kessler R.C. (2007). The prevalence and correlates of eating disorders in the National Comorbidity Survey Replication. Biol. Psychiatry.

[3] Marques L., Alegria M., Becker A.E., Chen C.N., Fang A., Chosak A., Diniz J.B. (2011). Compar-ative prevalence, correlates of impairment, and service utilization for eating disorders across US ethnic groups: Implications for reducing ethnic disparities in health care access for eating disorders. Int. J. Eat. Disord..

[4] Smink F.R., van Hoeken D., Oldehinkel A.J., Hoek H.W. (2014). Prevalence and severity of DSM-5 eating disorders in a community cohort of adolescents. Int. J. Eat. Disord..

[5] van Eeden A.E., van Hoeken D., Hoek H.W. (2021). Incidence, prevalence and mortality of anorexia nervosa and bulimia nervosa. Curr. Opin. Psychiatry.

[6] Ranzenhofer L.M., Jablonski M., Davis L., Posner J., Walsh B.T., Steinglass J.E. (2022). Early Course of Symptom Development in Anorexia Nervosa. J. Adolesc. Health.

[7] Khalsa S.S., Portnoff L.C., McCurdy-McKinnon D., Feusner J.D. (2017). What happens after treatment? A systematic review of relapse, remission, and recovery in anorexia nervosa. J. Eat. Disord..

[8] Miskovic-Wheatley J., Bryant E., Ong S.H., Vatter S., Le A., Touyz S., Maguire S., Consortium N.E.D.R. (2023). Eating disorder outcomes: Findings from a rapid review of over a decade of research. J. Eat Disord..

[9] Striegel-Moore R.H., Dohm F.A., Kraemer H.C., Taylor C.B., Daniels S., Crawford P.B., Schreiber G.B. (2003). Eating disorders in white and black women. Am. J. Psychiatry.

[10] Coleman K.J., Stewart C., Waitzfelder B.E., Zeber J.E., Morales L.S., Ahmed A.T., Ahmedani B.K., Beck A., Copeland L.A., Cummings J.R. (2016). Racial-Ethnic Differences in Psychiatric Diagnoses and Treatment Across 11 Health Care Systems in the Mental Health Research Network. Psychiatr. Serv..

[11] Schwartz R.C., Blankenship D.M. (2014). Racial disparities in psychotic disorder diagnosis: A review of empirical literature. World J. Psychiatry.

[12] Forcén F.E., Vélez Flórez M.C., Bido Medina R., Zambrano J., Pérez J.H., Rodríguez A.M., Santos L.H. (2023). Deconstructing Cultural Aspects of Mental Health Care in Hispanic/Latinx People. Psychiatr. Ann..

[13] Cheng Z.H., Perko V.L., Fuller-Marashi L., Gau J.M., Stice E. (2019). Ethnic differences in eating disorder prevalence, risk factors, and predictive effects of risk factors among young women. Eat. Behav..

[14] Howe C.P., Baik S.Y., D’Adamo L., Kouveliotes M., Pan Z., Monocello L., Firebaugh M.L., Eisenberg D., Newman M.G., Wilfley D.E. (2025). Examining Prevalence and Presentations of Eating Disorders Across Racial/Ethnic Groups in a National, Population-Based Sample of College Students. Int. J. Eat. Disord..

[15] Van Dyne A., Washington N., Villodas M., Cronan T. (2023). Racial/ethnic differences in anorexia and bulimia diagnoses among U.S. college students. Eat. Behav..

[16] Reba-Harrelson L., Von Holle A., Hamer R.M., Swann R., Reyes M.L., Bulik C.M. (2009). Patterns and prevalence of disordered eating and weight control behaviors in women ages 25-45. Eat. Weight. Disord..

[17] Swanson S.A., Crow S.J., Le Grange D., Swendsen J., Merikangas K.R. (2011). Prevalence and correlates of eating disorders in adolescents. Results from the national comorbidity survey replication adolescent supplement. Arch. Gen. Psychiatry.

[18] Udo T., Grilo C.M. (2018). Prevalence and Correlates of DSM-5-Defined Eating Disorders in a Nationally Representative Sample of U.S. Adults. Biol. Psychiatry.

[19] Zhang A.Y., Snowden L.R. (1999). Ethnic characteristics of mental disorders in five U.S. communities. Cultur. Divers. Ethn. Minor. Psychol..

[20] Alegria M., Woo M., Cao Z., Torres M., Meng X.L., Striegel-Moore R. (2007). Prevalence and correlates of eating disorders in Latinos in the United States. Int. J. Eat. Disord..

[21] Benjet C., Méndez E., Borges G., Medina-Mora M.E. (2012). Epidemiología de los trastornos de la conducta alimentaria en una muestra representativa de adolescentes. Salud Ment..

[22] Perez M., Ohrt T.K., Hoek H.W. (2016). Prevalence and treatment of eating disorders among Hispanics/Latino Americans in the United States. Curr. Opin. Psychiatry.

[23] Thomas J.J., Roberto C.A., Brownell K.D. (2009). Eighty-five per cent of what? Discrepancies in the weight cut-off for anorexia nervosa substantially affect the prevalence of underweight. Psychol. Med..

[24] Datta N., Hossepian K., Xie I., Gurcan H.Y., Behr S., Pouliadi M., Miranda C. (2024). Anorexia Nervosa Across the Lifespan: A Review of Recent Literature. Focus.

[25] Toppino F., Longo P., Martini M., Abbate-Daga G., Marzola E. (2022). Body Mass Index Specifiers in Anorexia Nervosa: Anything below the “Extreme”?. J. Clin. Med..

[26] Mustelin L., Silén Y., Raevuori A., Hoek H.W., Kaprio J., Keski-Rahkonen A. (2016). The DSM-5 diagnostic criteria for anorexia nervosa may change its population prevalence and prognostic value. J. Psychiatr. Res..

[27] Consultation W.E. (2004). Appropriate body-mass index for Asian populations and its implications for policy and intervention strategies. Lancet.

[28] Li Z., Daniel S., Fujioka K., Umashanker D. (2023). Obesity among Asian American people in the United States: A review. Obesity.

[29] Albrecht S.S., Gordon-Larsen P. (2013). Ethnic differences in body mass index trajectories from adolescence to adulthood: A focus on Hispanic and Asian subgroups in the United States. PLoS ONE.

[30] Howell C.R., Juarez L., Agne A.A., Nassel A.F., Scarinci I.C., Ayala G.X., Cherrington A.L. (2022). Assessing Hispanic/Latino and Non-Hispanic White Social Determinants of Obesity Among a Community Sample of Residents in the Rural Southeast US. J. Immigr. Minor. Health.

[31] Office of Minority Health Obesity and Hispanic Americans|Office of Minority Health. https://minorityhealth.hhs.gov/obesity-and-hispanic-americans.

[32] Forney K.J., Brown T.A., Holland-Carter L.A., Kennedy G.A., Keel P.K. (2017). Defining “significant weight loss” in atypical anorexia nervosa. Int. J. Eat. Disord..

[33] Harrop E.N., Mensinger J.L., Moore M., Lindhorst T. (2021). Restrictive eating disorders in higher weight persons: A systematic review of atypical anorexia nervosa prevalence and consecutive admission literature. Int. J. Eat. Disord..

[34] Kons K., Essayli J., Shook J. (2024). Comparing the knowledge, attitudes, and practices of pediatric and family medicine clinicians toward atypical anorexia nervosa versus anorexia nervosa. Int. J. Eat. Disord..

[35] Coffino J.A., Udo T., Grilo C.M. (2019). Rates of Help-Seeking in US Adults With Lifetime DSM-5 Eating Disorders: Prevalence Across Diagnoses and Differences by Sex and Ethnicity/Race. Mayo Clin. Proc..

[36] Cachelin F.M., Striegel-Moore R.H. (2006). Help seeking and barriers to treatment in a community sample of Mexican American and European American women with eating disorders. Int. J. Eat. Disord..

[37] Alegría M., Canino G., Ríos R., Vera M., Calderón J., Rusch D., Ortega A.N. (2002). Inequalities in use of specialty mental health services among Latinos, African Americans, and non-Latino whites. Psychiatr. Serv..

[38] Hochhausen L., Le H.N., Perry D.F. (2011). Community-based mental health service utilization among low-income Latina immigrants. Community Ment. Health J..

[39] Bazargan M., Cobb S., Assari S. (2021). Discrimination and Medical Mistrust in a Racially and Ethnically Diverse Sample of California Adults. Ann. Fam. Med..

[40] Cabassa L.J., Zayas L.H., Hansen M.C. (2006). Latino adults’ access to mental health care: A review of epidemiological studies. Adm. Policy Ment. Health.

[41] Maina I.W., Belton T.D., Ginzberg S., Singh A., Johnson T.J. (2018). A decade of studying implicit racial/ethnic bias in healthcare providers using the implicit association test. Soc. Sci. Med..

[42] Oakley L.P., López-Cevallos D.F., Harvey S.M. (2019). The Association of Cultural and Structural Factors with Perceived Medical Mistrust Among Young Adult Latinos in Rural Oregon. Behav. Med..

[43] Doshi M., Bryce R., Mesa H., Ibarra Avila M.C., Lopez W.D., Militzer M., Quinones S., Kraut R., Rion R., Fleming P.J. (2022). Barriers to health care services among US-based undocumented Latinx immigrants within the sociopolitical climate under the Trump administration: A comparative analysis between client experiences and provider perceptions. SSM Qual. Res. Health.

[44] Saunders J.F., Bravo E.I., Kassan A. (2023). “Anorexia doesn’t exist when you’re Latina”: Family, culture, and gendered expectations in eating disorder recovery. J. Latinx Psychol..

[45] McMiller W.P., Weisz J.R. (1996). Help-seeking preceding mental health clinic intake among African-American, Latino, and Caucasian youths. J. Am. Acad. Child. Adolesc. Psychiatry.

[46] Brelet L., Flaudias V., Désert M., Guillaume S., Llorca P.M., Boirie Y. (2021). Stigmatization toward People with Anorexia Nervosa, Bulimia Nervosa, and Binge Eating Disorder: A Scoping Review. Nutrients.

[47] Crisp A.H., Gelder M.G., Rix S., Meltzer H.I., Rowlands O.J. (2000). Stigmatisation of people with mental illnesses. Br. J. Psychiatry.

[48] Herbozo S., Stevens S.D., Moldovan C.P., Morrell H.E.R. (2017). Positive comments, negative outcomes? The potential downsides of appearance-related commentary in ethnically diverse women. Body Image.

[49] Eisenberg M.E., Berge J.M., Fulkerson J.A., Neumark-Sztainer D. (2011). Weight comments by family and significant others in young adulthood. Body Image.

[50] Ordaz D.L., Schaefer L.M., Choquette E., Schueler J., Wallace L., Thompson J.K. (2018). Thinness pressures in ethnically diverse college women in the United States. Body Image.

[51] Menon C.V., Harter S.L. (2012). Examining the impact of acculturative stress on body image disturbance among Hispanic college students. Cultur. Divers. Ethn. Minor. Psychol..

[52] Gordon K.H., Castro Y., Sitnikov L., Holm-Denoma J.M. (2010). Cultural body shape ideals and eating disorder symptoms among White, Latina, and Black college women. Cultur. Divers. Ethn. Minor. Psychol..

[53] Schaefer L.M., Burke N.L., Thompson J.K. (2019). Thin-ideal internalization: How much is too much?. Eat. Weight. Disord..

[54] Project HEAL Cost Of Eating Disorder Treatment. https://www.theprojectheal.org/cost-of-treatment.

[55] Guzman G. (2024). Median Income of Non-Hispanic White Households Increased While Asian, Black and Hispanic Median Household Income Did Not Change.

[56] Guzman G., Kollar M. (2024). Income in the United States: 2023.

[57] Scherer Z., Mayol-Garcia Y. (2022). Half of People of Dominican and Salvadoran Origin Experienced Material Hardship in 2020.

[58] Artiga S., Hill L., Damico A. Health Coverage by Race and Ethnicity, 2010–2022. https://www.kff.org/racial-equity-and-health-policy/issue-brief/health-coverage-by-race-and-ethnicity/.

[59] Kay J. 5 Things Everyone with an Eating Disorder Should Know About Their Insurance—National Eating Disorders Association. https://www.nationaleatingdisorders.org/5-things-everyone-eating-disorder-should-know-about-their-insurance/.

[60] Kritz F. How Much Does It Cost to Treat Bulimia, Anorexia, and Eating Disorders?. https://www.goodrx.com/conditions/eating-disorders/eating-disorders-treatment-costs?srsltid=AfmBOopztnfs37sqwtWibyAd91V2LXGWizYt6t4ckzMfU465P145bIY-.

[61] Kritz F. How Much Does It Cost to Treat Eating Disorders, and How Can I Pay for It?. https://www.goodrx.com/conditions/eating-disorders/eating-disorders-treatment-costs.

[62] Accurso E.C., Buckelew S.M., Snowden L.R. (2021). Youth Insured By Medicaid With Restrictive Eating Disorders-Underrecognized and Underresourced. JAMA Pediatr..

[63] Watson H.J., Yilmaz Z., Thornton L.M., Hübel C., Coleman J.R.I., Gaspar H.A., Bryois J., Hinney A., Leppä V.M., Mattheisen M. (2019). Genome-wide association study identifies eight risk loci and implicates metabo-psychiatric origins for anorexia nervosa. Nat. Genet..

