# Supplementary material for: Anorexia Nervosa in U.S. Hispanic/Latino Populations: Sociocultural and Structural Influences on Recognition and Care
Source: Nutrients. 2026 Oct 31;18(21):3622. doi: 10.3390/nu18213622 (PMC13766176; doi:10.3390/nu18213622)
Supplement: Supplementary file 1 [file nutrients-18-03622-s001.zip › nutrients-4598971-supplementary.pdf]

Table S1. Detailed sampling, diagnostic, and data-extraction characteristics of included studies						
**Study**	**Sampling description**	**Survey names**	**Sample sizes**	**Diagnostic definitions**	**BMI thresholds**	**Assessment administration**
**Cheng et al, 2019**	Combined data from one efficacy trial and two effectiveness trials. For the health prevention groups mailing and fliers were used to recruit female High School (trial 1 and 2) and college students (trial 1 and 3). In telephone screenings, participants were to report body image concerns.	Efficacy Trial 1: Dissonance and Healthy Weight Eating Disorder Prevention Programs: Long-Term Effects from a Randomized Efficacy Trial; Effectiveness Trial 2: An Effectiveness Trial of a Selected Dissonance-Based Eating Disorder Prevention Program for Female High School Students: Long-Term; Effectiveness Trial 3: Effectiveness Trial of a Selective Dissonance-Based Eating Disorder Prevention Program with Female College Students: Effects at 2- and 3-Year Follow-Up.	Total sample (N= 1177) ; White (n=848) ; Hispanic American (n=141); Asian American (n = 130); African American (n = 58)	DSM-5 eating disorders were assessed using the semi-structured Eating Disorder Diagnostic Interview (EDDI). The baseline assessment covered the preceding three months; follow-up assessments covered symptoms since the previous interview. Subthreshold AN was assessed rather than atypical AN because weight history was unavailable.	Specific Threshold was not reported.	Surveys and interviews were completed at baseline and at 1-, 6-, 12-, 24-, and 36- month follow-ups.
**Howe et al, 2025**	Students across 26 U.S. colleges/universities who received and completed a population-based online mental health screen by email. Eligible participants were ≥18 years. This email was either sent to the entire student body or a random subset, and either to undergraduate students from all years (17 schools) or only first- and second-year undergraduates (9 schools).	Stanford-Washington University Eating Disorders Screen (SWED).	Total sample (N=29,951); Asian (n = 4142); Black or African American (n= 1765); Hispanic (n = 4408); multiracial (n = 1535); White (n = 18,101)	SWED screening classification of probable AN based on self-reported BMI ≤18.45 and Weight Concerns Scale score ≥59.	Probable AN if BMI was ≤ 18.45 based on self-reported height/weight and a WCS score ≥ 59.	Online self-reported population-based screening survey.
**Marques et al, 2011**	Nationally representative U.S. adult (18 years or older) sample pooled from the NIMH CPES which combined NLAAS, NSAL, and NCS-R.	Collaborative Psychiatric Epidemiology Surveys (CPES); National Survey of American Life (NSAL); The National Latino and Asian American Study (NLAAS); and National Comorbidity Survey Replication (NCS-R).	NLAAS component: 2554 Latino and 2095 Asian American participants. The pooled analysis additionally included African American participants from NSAL and non-Latino White participants from NCS-R Part II; the combined analytic sample size and remaining subgroup sizes were not explicitly reported.	The presence of lifetime and past year diagnosis was assessed by the World Health Organization Composite International Diagnostic Interview (WMH-CIDI); DSM-IV	No specific threshold reported.	Fully structured, interview administered by WMH-CIDI.
**Reba-Harrelson et al., 2009**	Female U.S. residents ages 25-45 who received an email invitation and consented to an online "eating habits" survey. Participants identified using a national quota-sampling strategy that included stratification of 4 age groups: 25-29, 30-34, 35-39 and 40-45 (N=4023).	"Eating habits" survey.	Total N = 4023. Ethnicity: Hispanic/Latina, n = 539; non-Hispanic/Latina, n = 3484. Race: White, n = 3008; African American/Black, n = 544; Asian, n = 186; Native Hawaiian/Other Pacific Islander, n = 8, American Indian/Alaska Native, n = 36; Other, n = 241. Race and ethnicity were assessed separately; Hispanic/Latina participants may belong to any racial category.	Diagnostic questions were taken directly from studies with the Virginia Twin Registry; questions were modeled using the Structured Clinical Interview (SCID); DSM-IV; Assessed broadly defined AN. AN recall period not explicitly specified	BMI ≤18.5 and did not require amenorrhea.	Online self-reported survey.

**Swanson et al.,2011**	Combined nationally representative household (n = 879 adolescents) and school adolescent (n = 9244 adolescents) samples using the NCS-A (N=10,123), adolescents aged 13 - 18 years.	National Comorbidity Survey Replication Adolescent Supplement (NCS-A)	Total N = 10,123; household sample, n = 879; school sample, n = 9244.	DSM-IV disorders were assessed using the fully structured World Health Organization Composite International Diagnostic Interview (CIDI).	No specific threshold reported.	The fully structured interview was administered to adolescents by lay interviewers.
**Udo & Grilo, 2018**	A national sample of 36,306 U.S. adults that completed the NESARC-III. Respondents completed computer-assisted face-to-face personal interviews and were 18 years and older.	National Epidemiologic Survey on Alcohol and Related Conditions- III (NESARC-III)	Total N = 36,306	Used the National Institute on Alcohol Abuse and Alcoholism Alcohol Use Disorder and Associated Disabilities Interview Schedule-5 (AUDADIS-5) to assess DSM-5 disorders. To calculate 12 months and lifetime prevalence estimates, age of onset and age of most recent episode were used.	Self-reported lowest BMI < 18.5.	AUDADIS-5 was administered by 970 trained lay assessors who had 5 years of experience in related health surveys.
**Van Dyne, 2023**	U.S. college students (N = 426,425) who voluntarily completed the ACHA-NCHA IIC survey.	American College Health Association-National College Health Assessment II-C (ACHA-NCHA II-C).	Total N = 426,425. Reported racial/ethnic groups: White, n = 252,011; Black, n = 18,642; Hispanic/Latino, n = 43,743; Asian/Pacific Islander, n = 53,116; American Indian/Alaska Native/Native Hawaiian, n = 1923; Biracial/Multiracial, n = 43,929; Other, n = 6600. Participants categorized as Other were excluded from analysis.	"Within the last 12 months, have you been diagnosed or treated by a professional" for anorexia. Participants answered yes/no.	BMI was collected, but no BMI threshold was specified for the self-reported professional diagnosis/treatment outcome.	Self-reported (prior professional diagnosis/treatment) on college health survey .
**Zhang & Snowden, 1999**	Community residents from 5 U.S. Epidemiological Catchment Area sites. The Los Angeles site was analyzed separately. In all sites a multistage probability sampling design was used. One resident (18 years or older) was selected from each household.	Epidemiological Catchment Area (ECA)	Total Sample N = 18,152	The NIMH Diagnostic Interview Schedule (DIS) was used to assess lifetime prevalence; DSM-III	No specific threshold reported	The DIS was used to conduct interviews (designed for nonprofessional interviewers)
**Alegria, 2007**	Nationally representative U.S. Latino adult household sample from the NLAAS; Latinos aged 18 and older.	The National Latino and Asian American Study (NLAAS)	Total Sample: N = 2554.	The presence of lifetime and 12- month diagnosis was assessed using the diagnostic interview of the World Mental Health Composite International Diagnostic Interview (WMH-CIDI) ; DSM-IV	AN-specific numerical BMI threshold not explicitly reported; diagnosis followed DSM-IV–based WMH-CIDI algorithms referenced to Hudson et al.	Fully structured diagnostic interview conducted in English or Spanish.

Note. Race/ethnicity and sex/gender classifications were retained as defined in the original source studies. Categories may differ across studies in their definitions. Outcome periods and case ascertainment methods varied across studies. Abbreviations: ACHA-NCHA II-C = American College Health Association–National College Health Assessment II-C; AUDADIS-5 = Alcohol Use Disorder and Associated Disabilities Interview Schedule–5; CIDI = Composite International Diagnostic Interview; CPES = Collaborative Psychiatric Epidemiology Surveys; DSM-III = Diagnostic and Statistical Manual of Mental Disorders, Third Edition; DSM-IV = Diagnostic and Statistical Manual of Mental Disorders, Fourth Edition; DSM-5 = Diagnostic and Statistical Manual of Mental Disorders, Fifth Edition; ECA = Epidemiologic Catchment Area Study; EDDI = Eating Disorder Diagnostic Interview; NCS-A = National Comorbidity Survey Replication–Adolescent Supplement; NCS-R = National Comorbidity Survey Replication; NESARC-III = National Epidemiologic Survey on Alcohol and Related Conditions III; NLAAS = National Latino and Asian American Study; NSAL = National Survey of American Life; SWED = Stanford-Washington University Eating Disorders Screen; WMH-CIDI = World Mental Health Composite International Diagnostic Interview.

Table S2. Additional racial/ethnic anorexia nervosa estimates reported in included studies.

Study	Outcome type/ period	White estimate	Black/ African American estimates / NH Black	Asian/ PI estimates	Between group comparisons (effect estimates, 95% CI, p value)
Cheng et al., 2019	Study prevalence across baseline and 3-year follow-up.	6/848 (0.7%)	African American: 0/58 (0.0%)	Asian American: 1/130 (0.8%)	3-year AN onset, African American vs. White (ref.): HR= 0.96 (95% CI 0.13-7.41); P= 0.971.<br>3-year AN onset Asian American vs. White (ref.): HR= 0.43 (95% CI 0.16-1.14); P= 0.091.
Howe et al., 2025	Screening prevalence of probable AN	Cisgender Women: 243/10072 (2.41%); Cisgender Men: 21/5133 (0.41%); Non-cisgender: 9/590 (1.53%)	Cisgender Women: 6/1073 (0.56%); Cisgender Men: 0/323 (0.00%); Non-cisgender: 0/47 (0.00%)	Cisgender Women: 78/2161 (3.61%); Cisgender Men: 8/1285 (0.62%); Non-cisgender: 7/81 (8.64%)	No statistical detail reported for comparisons.
Marques et al., 2011	Lifetime weighted prevalence	Non-Latino White All 0.39%; female: 0.64%; male : 0.14%	African American All: 0.15%; female: 0.12%; male: 0.18%	Asian All: 0.10%; female: 0.13%; male: 0.07%	(African American vs White) All: *P*=0.1377; (Asian vs White) All: *P*=0.0797
	12-month weighted prevalence	All: 0.03%; female: 0.02%; male: 0.04%	African American All: 0.06%; female: 0.01%; male: 0.10%	Asian All: 0.05%; female: 0.04%; male: 0.07%	(African American vs White) All: *P*=0.6369; (Asian vs White) All: *P*=0.7582
Reba-Harrelson et al., 2009	Weighted prevalence of broadly defined AN; recall period not explicitly specified	Weighted prevalence: 0.3%	African American/Black weighted prevalence: 0%	Asian weighted prevalence: 0%<br>Hawaiian/Pacific Islander: 0%	No statistical detail reported for comparisons.
Swanson et al., 2011	Lifetime prevalence	Non-Hispanic white: 0.4%	Non-Hispanic Black: 0.1%	Not reported	No statistical detail reported for comparisons.
Udo & Grilo, 2018	Lifetime weighted prevalence	Non-Hispanic white: 206 cases (0.96%)	Non-Hispanic Black: 17 cases (0.19%)	Not Reported	Non-Hispanic Black vs. non-Hispanic White (ref.): AOR= 0.19 (95% CI 0.11-0.33); P<0.01.
	12-month weighted prevalence	Non-Hispanic white: 11 cases (0.07%)	Non-Hispanic Black: 0 cases (0.0%)		Not reported
Van Dyne, 2023	Past-year self-reported professional diagnosis/treatment	Not Reported	Not Reported	Not Reported	Black vs White (ref.) OR= 0.572 (95% CI 0.485-0.675); P<0.001<br>Asian/Pacific Islander vs White (ref.) OR= 0.653 (95% CI 0.598-0.713); P<0.001

Zhang & Snowden, 1999	Lifetime weighted prevalence	Full sample: 0.9%	Black full sample: 0.4%	Asian full sample: 0.6%	Black vs White (ref.) AOR=0.372 (95% CI 0.194-0.716); P ≤ .01<br>Asian vs White (ref.) AOR=0.606 (95% CI 0.095-3.861); P value not reported
Alegría et al., 2007	Lifetime and 12-month Bayesian prevalence	Not Reported	Not Reported	Not Reported	Not Applicable

Note. Race/ethnicity and sex/gender classifications were retained as defined in the original source studies. The combined Asian/Pacific Islander category in Van Dyne et al. (2023) reflects the source study's classification. Categories may differ across studies in their definitions. Sex and gender classifications were retained as reported in the original studies and should not be interpreted as directly equivalent. Outcome periods and case ascertainment methods varied across studies. Abbreviations: AOR: adjusted odds ratio; HR = hazard ratio; CI = confidence interval; ref. = reference group.

Table S3. Database-specific search strategies, final search dates, and filters/limits

Database	Final search date	Full search string	Filter/limits
PubMed	11-Aug-26	**1st search**: ("Anorexia Nervosa"[Mesh] OR "anorexia nervosa"[Title/Abstract]) AND (Hispanic*[Title/Abstract] OR Latino*[Title/Abstract] OR Latina*[Title/Abstract] OR Latinx[Title/Abstract])	No database filters applied.
		**2nd search**: ("Anorexia Nervosa"[Mesh] OR "anorexia nervosa"[Title/Abstract]) AND (race[Title/Abstract] OR racial[Title/Abstract] OR ethnic*[Title/Abstract] OR minority[Title/Abstract] OR minorities[Title/Abstract])	
		**3rd search:** ("Anorexia Nervosa"[Mesh] OR "anorexia nervosa"[Title/Abstract]) AND (Hispanic*[Title/Abstract] OR Latino*[Title/Abstract] OR Latina*[Title/Abstract] OR Latinx[Title/Abstract]) AND (prevalence[Title/Abstract] OR incidence[Title/Abstract] OR epidemiology[Title/Abstract])	
Web of Science	11-Aug-26	**1st search**: ("anorexia nervosa" AND (Hispanic* OR Latino* OR Latina* OR Latinx))	Searched in "Web of Science Core Collection"; Under editions "all" was selected; and under fielded search "Topic" was selected.
		**2nd search**: "anorexia nervosa" AND (race OR racial OR ethnic* OR minority OR minorities)	
		**3rd search:** "anorexia nervosa" AND (Hispanic* OR Latino* OR Latina* OR Latinx) AND (prevalence OR incidence)	

Google Scholar	11-Aug-26	"Eating disorder" Hispanic Latino prevalence anorexia	The first 110 results ranked by relevance were screened. Screening stopped because subsequent results were increasingly duplicative or unrelated to the review topic. This limit may have resulted in missed eligible studies.
